# Outdoor air pollution and respiratory health: a bibliometric analysis of publications in peer-reviewed journals (1900 – 2017)

**DOI:** 10.1186/s40248-018-0128-5

**Published:** 2018-06-01

**Authors:** Waleed M. Sweileh, Samah W. Al-Jabi, Sa’ed H. Zyoud, Ansam F. Sawalha

**Affiliations:** 1 Division of Biomedical Sciences, Department of Physiology, Pharmacology and Toxicology, College of Medicine and Health Sciences, An-Najah National University, Nablus, Palestine; 2 Department of Clinical and Community Pharmacy, College of Medicine and Health Sciences, An-Najah National University, Nablus, Palestine

**Keywords:** Outdoor air pollution, Respiratory health, Bibliometric analysis

## Abstract

**Background:**

Outdoor air pollution is a major threat to global public health that needs responsible participation of researchers at all levels. Assessing research output is an important step in highlighting national and international contribution and collaboration in a certain field. Therefore, the aim of this study was to analyze globally-published literature in outdoor air pollution – related respiratory health.

**Method:**

Outdoor air pollution documents related to respiratory health were retrieved from Scopus database. The study period was up to 2017. Mapping of author keywords was carried out using VOSviewer 1.6.6.

**Results:**

Search query yielded 3635 documents with an *h*-index of 137. There was a dramatic increase in the number of publications in the last decade of the study period. The most frequently encountered author keywords were: air pollution (835 occurrences), asthma (502 occurrences), particulate matter (198 occurrences), and children (203 occurrences). The United States of America ranked first (1082; 29.8%) followed by the United Kingdom (279; 7.7%) and Italy (198; 5.4%). Annual research productivity stratified by income and population size indicated that China ranked first (22.2) followed by the USA (18.8). Analysis of regional distribution of publications indicated that the Mediterranean, African, and South-East Asia regions had the least contribution. Harvard University (92; 2.5%) was the most active institution/organization followed the US Environmental Protection Agency (89; 2.4%). International collaboration was restricted to three regions: Northern America, Europe, and Asia. The top ten preferred journals were in the field of environmental health and respiratory health. *Environmental Health Perspective* was the most preferred journal for publishing documents in outdoor pollution in relation to respiratory health.

**Conclusion:**

Research on the impact of outdoor air pollution on respiratory health had accelerated lately and is receiving a lot of interest. Global research networks that include countries with high level of pollution and limited resources are highly needed to create public opinion in favor of minimizing outdoor air pollution and investing in green technologies.

**Electronic supplementary material:**

The online version of this article (10.1186/s40248-018-0128-5) contains supplementary material, which is available to authorized users.

## Background

Outdoor air pollution is defined as the presence of one or more substances in the atmospheric air at concentrations and duration above the natural limits [1]. Such substances include ozone [O3], airborne lead [Pb], carbon monoxide [CO], sulphur oxides [SOx] and nitrogen oxides [NOx] [2]. Recently, air pollution with particulate matters (PM), especially those with less than 2.5 μm, has been the focus of most outdoor air pollution studies due to its ability to penetrate the lung tissue and induce local and systemic effects [2].

Air pollution has been described as one of the “great killers of our age” and as “major threat to health” due to its tremendous and various health effects on humans of all ages and in both genders [3, 4]. In 2014, the World Health Organization (WHO) estimated that 92% of the world population was living in places with less than optimum outdoor air quality. Furthermore, WHO reported that in 2012, outdoor air pollution caused around 3 million deaths worldwide and 6.5 million deaths (11.6% of all global deaths) were associated with indoor and outdoor air pollution together [5].

Air pollution was linked to cancer, respiratory diseases, negative pregnancy outcomes, infertility, cardiovascular diseases, stroke, cognitive decline, and other adverse medical conditions [6–13]. Nearly 90% of air-pollution-related deaths occur in low- and middle-income countries, with nearly 2 out of 3 occurring in South-East Asia and Western Pacific regions. The problem of outdoor pollution is not a new one, but the rapid urbanization, particularly in Asia, made the problem of air pollution more visible and its health burden more tangible [14–17].

Bibliometric analysis is the application of statistical methods on published literature to analyze publication trends with time and to shed light on influential researchers, countries, and institutions in the field. In the past decade, at least seven bibliometric studies on air pollution were published [18–24]. However, none of the published bibliometric studies have shed light on the air pollution - related respiratory health. Therefore, in the current study, we aim to analyze literature pertaining to outdoor air pollution and respiratory health. The size of the literature and research productivity in this field is a good indicator of national and international efforts to improve air quality and to decrease the health and economic burden of air pollution. Furthermore, the quality of the air we breathe is the responsibility of everyone including researchers and academics. This study comes in line with perceived personal responsibility toward better air quality.

## Method

### Search strategy

This study aimed to analyze the documents about outdoor air pollution – related respiratory health. Scopus database was used to retrieve relevant documents because of its advantages over other databases [25–28]. The search strategy developed for this study consisted of nine steps (Additional file 1). The first six steps utilized various keywords and search queries to retrieve the maximum number of documents. Keywords included in search queries were those found in recent relevant systematic reviews [6, 12, 13, 29, 30]. The combined result of search queries underwent a filtration process by adding exclusion and limitation components (steps 7 – 9).

False positive results were minimized by using title search. Therefore, all retrieved documents have the keywords of interest. Despite that, false positive results need to be searched by reviewing the retrieved documents. The review process was carried out on a sample of 200 documents chosen based on the number of citation. The review process was carried out by the authors (W.S and A.J) and approved by a third author (A.S). Keywords of the irrelevant documents (false positive results) were used in the exclusion step. A complete list of irrelevant keywords is written in Additional file 1. The exclusion of false positive results is not enough to confirm the validity of the search strategy. Therefore, the authors compared two different methods of data collection. In the first one, we collected data regarding research output for each of the most active authors as obtained through the search strategy, whilst in the second one, the research output of each of the most active authors was extracted and reviewed by exploring the author profile as presented by Scopus. The extent of agreement between the two methods is measured by interclass correlation coefficient using SPSS [31–35]. An excellent agreement between the two methods with an interclass correlation above 95% and a *p* less than 5% is indicative of high validity of the search strategy. In the current study, the interclass correlation was 0.98.7% and *p* was 0.001.

The retrieved data were also sorted based on the number of different country affiliations per article to calculate international collaboration. Documents with authors from different countries represent international or inter-country collaboration while documents in which all authors have one country affiliation represent intra-country collaboration. It should be emphasized that Scopus has the function which can separate documents with intra or inter – country collaboration. Therefore, the calculation of international collaboration was extracted from data provided by Scopus.

### Bibliometric analysis versus systematic reviews

It should be emphasized that the bibliometric analysis is not the same as systematic reviews. In contrast to systematic reviews, bibliometric analysis focuses on quantitative and qualitative aspects of all documents retrieved from one electronic large database. In bibliometric analysis, the investigated research question is the volume of research published, how this volume of literature evolved with time, what major topics were of high interest, and the scientific impact of literature in a particular subject. However, in systematic reviews, a complete and exhaustive summary of current literature obtained from several electronic databases and relevant to a research question is provided.

In bibliometric analysis, only one large and well – known database, such as Scopus, is used. Therefore, the retrieved documents will not include any duplicates. On the other hand, duplicate documents might appear in systematic reviews because several databases are used to retrieve the required documents.

### Data analysis and visualization

In this study, Hirsch-index (*h*-index) was used as a measure of impact of publications [36]. Graphs were created using Statistical Package for Social Sciences (SPSS). Hirsch - index is defined as the number of articles (n) that have received at least n citations [36]. VOSviewer software was used to create visualization maps while ArcMap 10.1 was used to create geographical distribution of the retrieved documents [37–39]. For VOSviewer mapping of most frequent author keywords, a minimum occurrence of 10 was used as a cut-off point for inclusion of the keyword in mapping analysis. Analysis also included distribution of publications based on World Health Organization (WHO) regions.

## Results

### Types and growth of publications

**Fig. 1:**
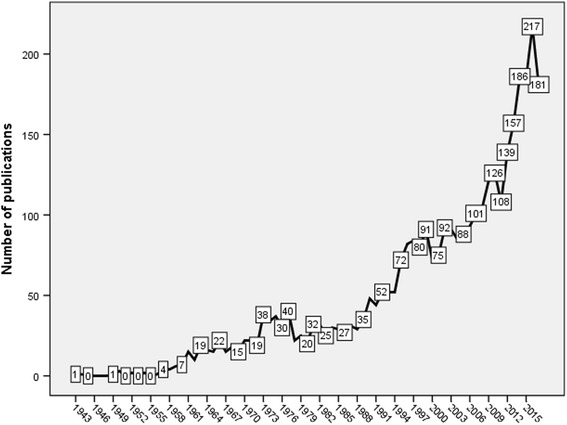
Annual growth of publications in Outdoor air pollution and respiratory health (1900 – 2017)

The search strategy yielded 3635 documents. The earliest document in this field was published in 1943 in *American Journal of Epidemiology* [40]. The analysis of the types of documents showed that research articles (2935, 80.7%) were the most common type followed by review articles (359; 9.9%). The remaining documents (341; 9.4%) were conference papers, letters, editorials, short surveys, and notes. English (2923, 80.4%) was the primary language of documents followed by French (156; 4.3%) and German (124; 3.4%). The subject areas of the documents were medicine (2772; 76.3%) followed by environmental science (1038; 28.6%) and biochemistry/ genetics/ molecular biology (317; 8.7%) with the possibility of overlap among different subject areas. The growth of publications showed a dramatic increase in the past decade. Figure 1 shows the annual growth of publications. There was a 72% increase in number of publications in 2017 compared to that in 2008.

### Author keywords

**Fig. 2:**
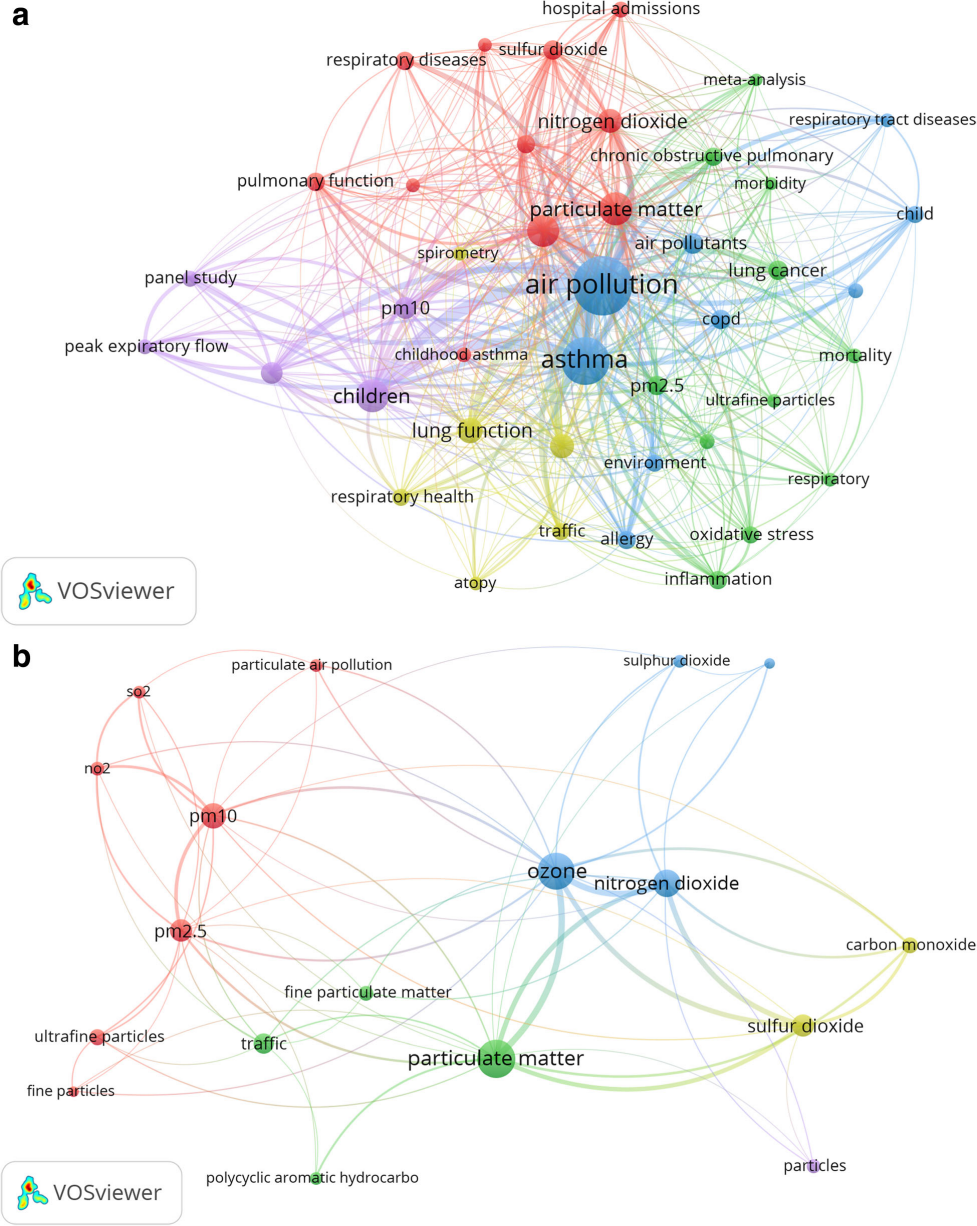
Most frequent author keywords encountered in the retrieved documents (**a**) and most frequently encountered types of outdoor air pollutants encountered in the retrieved documents (**b**)

Analysis of author keywords showed that the most frequently encountered author keywords were: air pollution (835 occurrences), asthma (502 occurrences), particulate matter (198 occurrences), and children (203 occurrences) (Fig. 2a). Further mapping of types of pollutants most commonly encountered in author keywords showed that particulate matter (198 occurrences), ozone (192 occurrences), nitrogen oxide (95 occurrences), PM10 (75 occurrences), PM2.5 (57 occurrences), and Sulfur dioxide (54 occurrences), were the most frequently encountered author keywords (Fig. 2b).

### Active journals

Table 1 shows the top ten journals that were involved in publishing the retrieved documents. *Environmental Health Perspective* was the most active journal (153; 4.2%) followed by *Environmental Research* (112; 3.1%) and *American Journal of Respiratory and Critical Care Medicine* (100; 2.8%). The top ten active journals included four in the field of environmental health, four in the field of respiratory health, one in allergy/immunology, and the last one in toxicology field.

**Table 1:** Top active journals in publishing documents in air pollution – related respiratory health (1900 – 2017)

Journal	Frequency	%
		*N* = 3635
Environmental Health Perspectives	153	4.2
Environmental Research	112	3.1
American Journal Of Respiratory And Critical Care Medicine	100	2.8
European Respiratory Journal	90	2.5
Archives Of Environmental Health	89	2.4
Thorax	58	1.6
Journal Of Allergy And Clinical Immunology	54	1.5
American Review Of Respiratory Disease	52	1.4
Inhalation Toxicology	52	1.4
International Journal Of Environmental Research And Public Health	41	1.1

### Authorship analysis

**Fig. 3:**
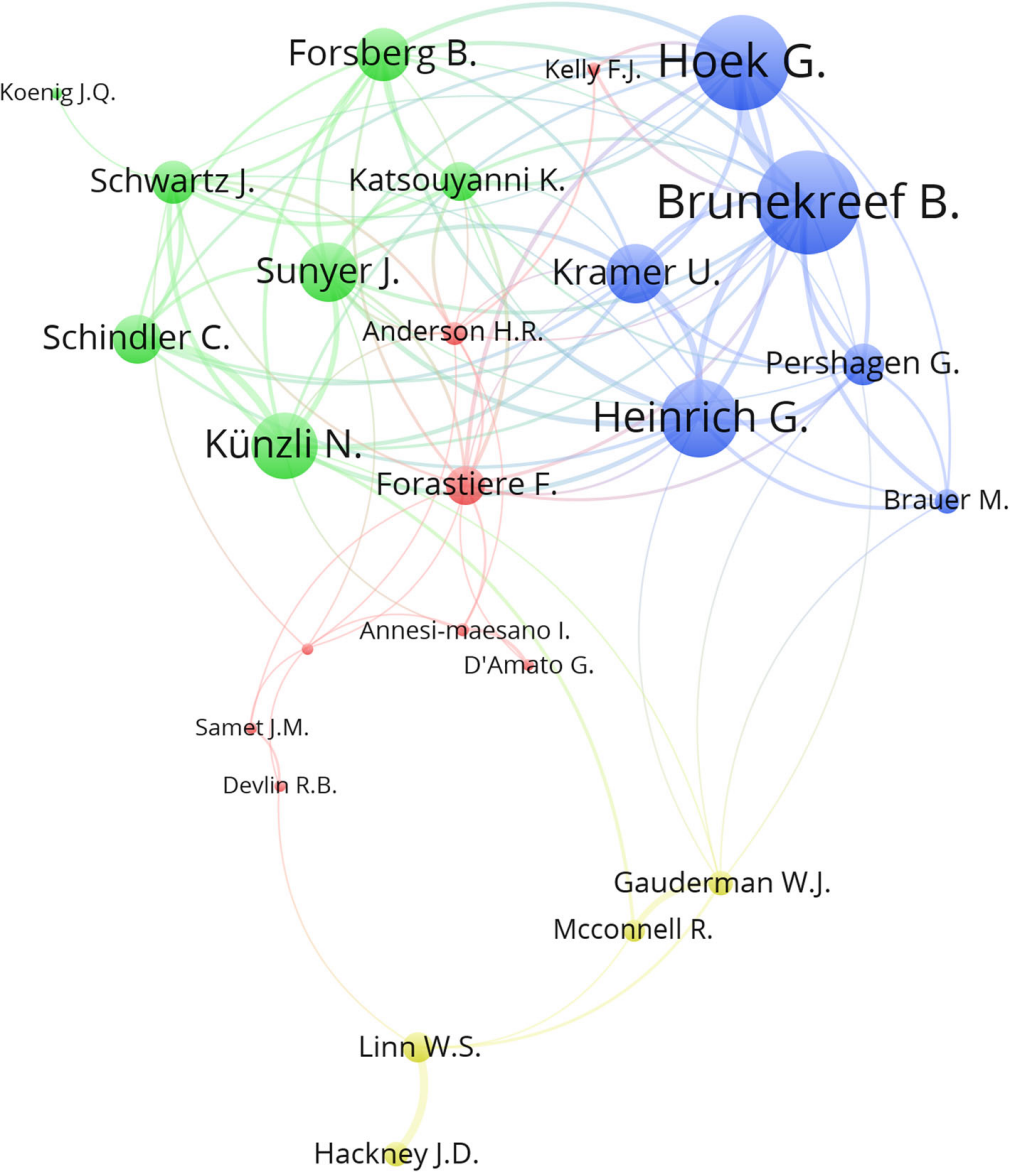
Network visualization map of authors with minimum productivity of 20 publications in the studied field and exist within a collaborative research group

The number of different author names who participated in publishing documents was 11,014; giving an average of 3.0 authors per document. Table 2 lists the top ten active authors with their affiliations. The top active authors were mainly from Western and Northern Europe, particularly from the Netherlands, Italy, and the United Kingdom [21]. Prof. Brunekreef, B. from the Netherlands was the most active researcher in this field with 77 (2.2%) documents. Authors with a minimum productivity of 20 publications were also visualized using network visualization map that showed research networking among active authors (Fig. 3). The map showed that active authors with minimum productivity of 20 publications existed in four clusters. The largest cluster consisted of eight authors (dark red color). The second cluster consisted of seven authors (green). The third cluster consisted of six authors (blue). The fourth cluster consisted of four researchers (dark yellow). Authors with minimum productivity of 20 publications who are not shown in the map are usually those who did not exist within a research network that has prominent productivity.

**Table 2:** Most active researchers in the field of air pollution – related respiratory health (1900 – 2017)

Rank	Researcher	Frequency	%	Affiliation
			*N* = 3635	
1st	Brunekreef, B.	77	2.2	Utrecht University, Utrecht, Netherlands
2nd	Hoek, G.	62	1.7	Utrecht University, Institute for Risk Assessment Sciences (IRAS), Utrecht, Netherlands
3rd	Schwartz, J.	42	1.2	Harvard School of Public Health, Department of Environmental Health, Boston, United States
4th	Künzli, N.	38	1.0	Swiss Tropical and Public Health Institute (Swiss TPH), Basel, Switzerland
5th	Heinrich, J.	36	1.0	Ludwig-Maximilians-Universitat Munchen, Social and Environmental Medicine, Munich, Germany
6th	Sunyer, J.	33	0.9	Instituto de Salud Global de Barcelona, Barcelona, Spain
7th	Linn, W.S.	33	0.9	University of Southern California, Department of Preventive Medicine, Los Angeles, United States
8th	Forastiere, F.	29	0.8	Lazio Regional Health Service, Department of Epidemiology, Roma, Italy
9th	Koenig, J.Q.	28	0.8	No affiliation available in Scopus
10th	Kelly, F.J.	28	0.8	King’s College London, Faculty of Life Sciences and Medicine, London, United Kingdom

### Active countries

**Fig. 4:**
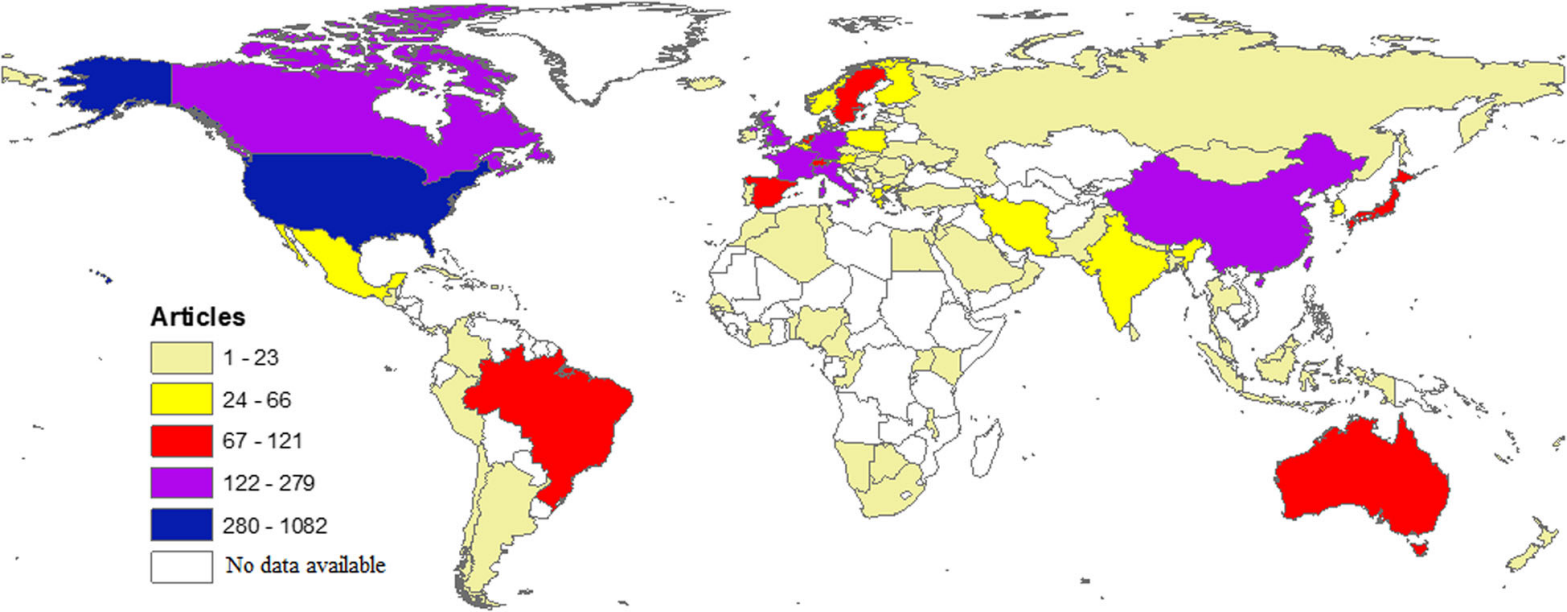
Geographical distribution of published research in outdoor air pollution and respiratory health (1900 – 2017)

**Fig. 5:**
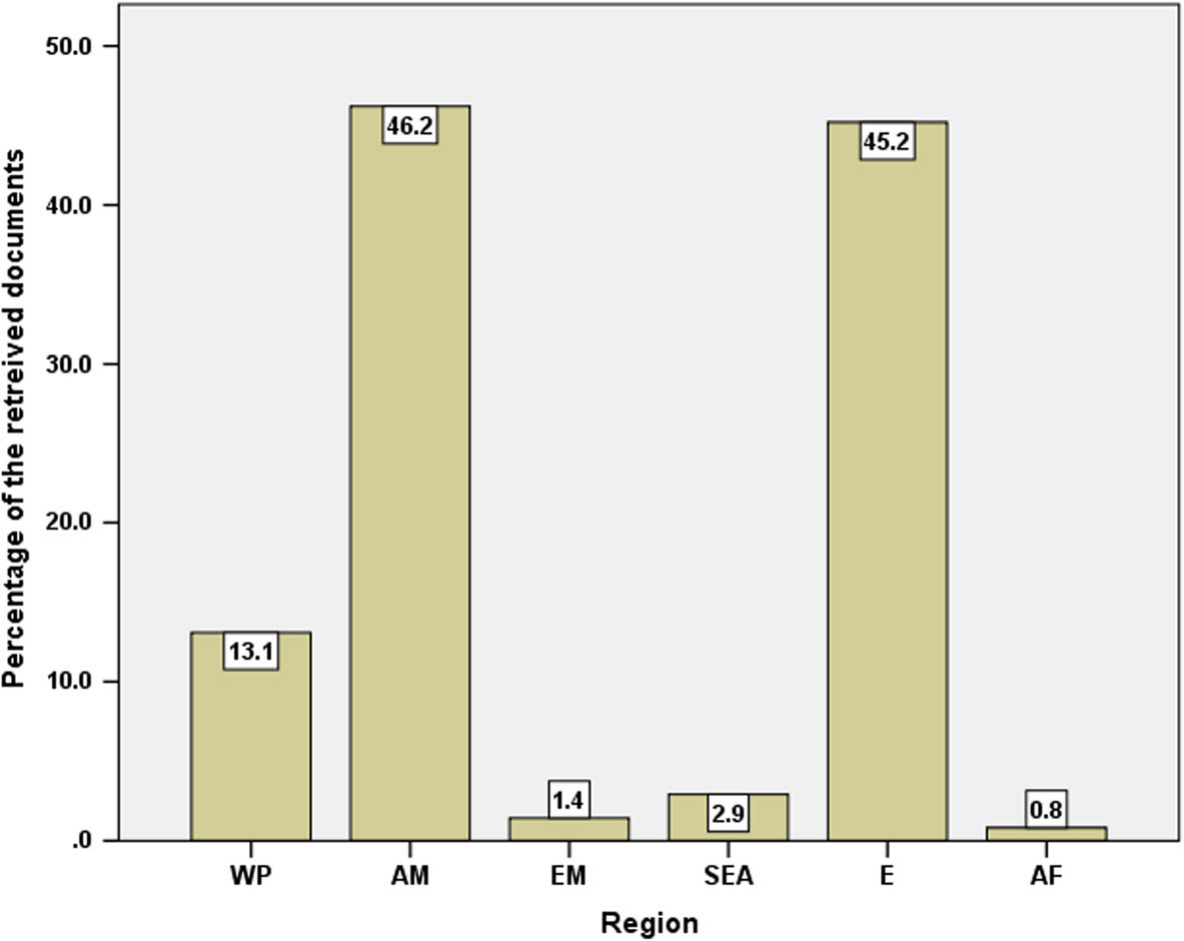
Geographical distribution of published research in outdoor air pollution and respiratory health (1900 – 2017) based on WHO world region. WP: Western Pacific; EM: Eastern Mediterranean; E: Europe; SEA: South Eastern Asia; AM: Americas; AF: Africa

Researchers from 92 different countries contributed to the retrieved documents. Table 3 lists the top ten countries actively involved in air pollution – related respiratory health. Researchers from the USA participated in publishing 1082 (29.8%) documents. The top 10 list included countries from Northern America, Western Europe, and Asia. Researchers from these top ten countries participated in publishing 2630 (72.3%) documents. Figure 4 shows worldwide geographical distribution of retrieved documents. Regional distribution of retrieved documents indicated that the regions of Americas, Europe, and Western pacific had the highest percentage of contribution while Mediterranean, Africa, and South-East Asia regions had the least contribution (Fig. 5).

**Table 3:** Top 10 active countries in publishing documents in air pollution – related respiratory health (1900 – 2017)

Country	Frequency (%)	GDP per capita	Productivity per GDP/capita/year	Intra-country collaboration	Inter – country collaboration^a^
	*N* = 3635	(^a^ 10^3^) USD per year			
United States	1082 (29.8)	57.467	18.8	806 (74.5)	276 (25.5)
United Kingdom	279 (7.7)	39.899	7.0	152 (54.5)	127 (45.5)
Italy	198 (5.4)	30.527	6.5	131 (66.2)	67 (33.8)
France	196 (5.4)	36.855	5.3	130 (66.3)	66 (33.7)
China	180 (5.0)	8.123	22.2	90 (50.0)	90 (50.0)
Canada	169 (4.6)	42.158	4.0	93 (55.0)	76 (45.0)
Germany	169 (4.6)	41.936	4.0	101 (59.8)	68 (40.2)
Netherlands	121 (3.3)	45.295	2.7	55 (45.5)	66 (54.5)
Sweden	119 (3.3)	51.600	2.3	50 (42.0)	69 (58.0)
Japan	117 (3.2)	38.895	3.0	91 (77.8)	26 (22.2)

^a^Inter – country collaboration = international collaboration

### International collaboration

**Fig. 6:**
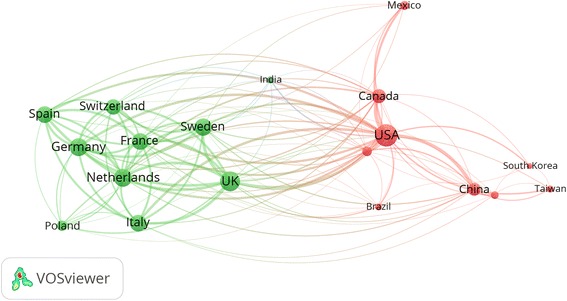
Network visualization map of research collaboration in outdoor air pollution and respiratory health (1900 – 2017)

International collaboration in air pollution – related respiratory health showed that there were three clusters. There was relatively adequate collaboration among countries within each cluster and there was adequate collaboration between countries in the two different clusters (Fig. 6). The first cluster consisted of 9 European countries shown in green color while the second cluster consisted of 9 countries in different regions in the world particularly those in Northern and Southern America, South East Asia, and Western Pacific regions. The third cluster consisted of one item, India with research connections with countries in both cluster number 1 and 2. International collaboration among countries in the Mediterranean region, Africa, or Eastern Europe and those in Northern America, Europe, or Asia did not show up in the map.

Table 3 shows the extent and the percentage of intra and inter (international) country collaboration for the top active countries. In terms of quantity, the USA had the largest number of documents (276; 25.5%) with international authors. However, this quantity represents only 25.5% of total research productivity from the USA which means that approximately 75% of USA research production in this field was produced by authors from the USA without collaboration with international researchers. Japan had the least percentage (22.2%) of international collaboration while Sweden had the largest percentage (58.0%) of documents with international collaboration.

### Active institutions

Harvard University ranked first in research output (92; 2.5%) and in the impact of publications (*h*-index = 44). The US Environmental Protection Agency (EPA) ranked second in research output (89; 2.4%) and in the impact of publications (*h*-index = 36). Table 4 shows the top ten active institutions/organizations. The list included seven academic institutions and three research centers. Six of the top active institutions were American institutions, three were European, and one was Canadian.

**Table 4:** Top active institutions/organizations in publishing documents in air pollution – related respiratory health (1900 – 2017)

Institution/Organization (country affiliation)	Frequency	%	*h*-index
		*N* = 3635	
Harvard School of Public Health	92	2.5	44
United States Environmental Protection Agency	89	2.4	36
University of Southern California	65	1.8	32
The University of North Carolina at Chapel Hill	64	1.8	26
University of Washington, Seattle	61	1.7	28
Inserm	60	1.7	24
The University of British Columbia	58	1.6	30
Utrecht University	53	1.5	29
Helmholtz Center Munich German Research Center for Environmental Health	52	1.4	29
Universitat Basel	52	1.4	23

### Citation analysis

The total number of citations received by documents was 101,113, with an average of 27.8 citations per document. Range of citations was [0 – 4294]. The *h*-index of the retrieved documents was 137. Table 5 shows the top ten highly cited articles. The article that received the highest number of citations (4294) was published in 2002 in *Journal of American Medical Association* (JAMA) and discussed the relation between lung cancer, cardiopulmonary mortality and air pollution [40].

**Table 5:** Top 10 cited documents in air pollution – related respiratory health (1900 – 2017)

Reference	Title	Cited by
[80]	“Lung cancer, cardiopulmonary mortality, and long-term exposure to fine particulate air pollution”	4294
[81]	“Acute respiratory effects of particulate air pollution”	1561
[82]	“Fine particulate air pollution and hospital admission for cardiovascular and respiratory diseases”	1088
[83]	“Respiratory effects are associated with the number of ultrafine particles”	975
[84]	“Pulmonary effects of inhaled ultrafine particles”	855
[85]	“The effect of air pollution on lung development from 10 to 18 years of age”	729
[86]	“Particulate air pollution and hospital emergency room visits for asthma in Seattle”	510
[87]	“Air pollution from truck traffic and lung function in children living near motorways”	467
[88]	“Acute effects of particulate air pollution on respiratory admissions: Results from APHEA 2 project”	465
[89]	“Asthma in exercising children exposed to ozone: A cohort study”	464

## Discussion

### Growth of publications

In this study, we analyzed global research output in outdoor air pollution – related respiratory health. The results showed a noticeable increase in the number of publications in the last decade of the study period. This indicates that the level of air pollution and its health consequences reached serious levels. In 2012, air pollution was responsible for 3 million deaths, representing 5.4% of the total global deaths. In the same year, about 25% were due to lung cancer deaths, 8% were due to chronic obstructive pulmonary disease (COPD) deaths, and about 17% of respiratory infection deaths were caused by outdoor air pollution [41]. A study indicated that the contribution of outdoor air pollution to global premature mortality could double by 2050 [42]. Another study concluded that outdoor air pollution contributes to the increase in global burden of COPD and that an increase of 10 μg/m^(3)^ in PM10 produced significant increase in COPD deaths and exacerbations that can be substantially reduced by controlling air pollution [43]. A cohort Chinese study concluded that the risks of mortality and years of life lost were elevated corresponding to an increase in current ambient concentrations of the air pollutants [44].

The contribution of researchers from /ifferent scientific fields led to an acceleration in the growth of publications in this field. Scientists in the fields of the environment, respiratory health, public health, and even molecular biology/genetics contributed to the retrieved documents [45–49]. The fact that air pollution is a multidisciplinary field created a large number of readers from different scientific fields and thus leading to large number of citations, reflected in the relatively high *h*-index value of documents. For example, the *h*-index of literature in global carbapenem resistance was 102 and that for literature in resistant tuberculosis was 76 [50, 51].

### Active countries and institutions

Our results showed that China had the highest research productivity in terms of GDP per capita per year. In China, air pollution was previously estimated to contribute to 1.2 to 2 million deaths annually [52]. In its list of the world’s deadliest countries for air pollution, the WHO ranked China first followed by India, Russian Federation, Indonesia, Pakistan, Ukraine, Nigeria, Egypt, USA, and Bangladesh [53]. Out of the top 10 countries that have high total annual number of deaths from PM2.5 and PM10, only China and USA were among the top ten active countries in research output. The deadliest effects of air pollution in China led to the adoption of the Ambient Air Quality Standard in China in 2012 [54]. This system started a national Air Reporting System that now includes 945 sites in 190 cities.

The presence of active institutions and many high impact journals in the field of environmental health and respiratory medicine issued from the USA contributed to the leadership of USA in this field. Research output in any field is a function of money allocated to research as well as public health agendas of the country. The leadership of the USA was seen in several other scientific subjects [55–58]. The fact that English was the primary language of literature published in journals indexed in Scopus might have created some sort of bias toward English-speaking countries.

### International collaboration

Outdoor air pollution is a global health concern and international collaboration in this field is necessary. In our study, the extent of international collaboration in research was relatively high, particularly within European countries and between USA and Asian countries. The WHO Collaborating Centre for Air Quality Management and Air Pollution Control (WHO CC) is working with member states in Europe and Asia to encourage collaboration in air quality programs through interaction and networking [59].

### Highly cited documents

The top cited documents in the field was about the relationship between outdoor air pollution and lung cancer; and received a large number of citations suggestive of great importance. The *International Agency for Research on Cancer* [60], which is part of the WHO, has classified outdoor air pollution, as a whole, as a cancer-causing agent (carcinogen) [60]. The International Agency for Research on Cancer (IARC) concluded that outdoor air pollution causes lung cancer and is associated with increased risk for bladder cancer. Urgent action to minimize level of outdoor air pollution and exposure of population to such carcinogenic pollutants is necessary, particularly in cities with high levels of outdoor air pollution [61, 62].

### Strength and limitations

It is the first to assess research activity in the field of outdoor air pollution – related respiratory health. Our study documented the accelerated increase in publications and the role of international collaboration. However, our study has a number of limitations. The Scopus database is a comprehensive and large database that includes different disciplines, but some peer – reviewed journals are not indexed in Scopus. This is particularly true for journals published from India, China, Indonesia, and other Asian and African countries where outdoor air pollution is a real public health problem. Therefore, documents published in un-indexed journals were not retrieved. Secondly, the results presented in this study reflect the search strategy implemented which is comprehensive in the subject but the presence of false positive and false negative results cannot be ruled out. This is true in all bibliometric studies [63–67]. Thirdly, when listing active authors and institutions, the authors depended on the outcome obtained from the Scopus. However, some authors might have more than one Scopus profile or might have their name written in different articles in different ways which will affect their research output and therefore their rank as well. Same applies to active institutions where the name of the institution might be written in different articles in different ways which will affect their research output and rank as well. Furthermore, the authors used the keyword “environment” in the search strategy in a strict way to avoid false positive results since not all environmental pollution could fit the scope of the current study which focused on outdoor air pollution and its impact on respiratory health. In this regard, the authors also avoided the use of the keyword “climate” in the search strategy to keep the research question focused on outdoor air pollution, particularly those produced by industry. Finally, it should be emphasized that the list of highly cited articles does not mean that these articles are the only influential ones in the field. The citation process is dynamic and sometimes high citation reflects self-citation rather than impact. There were many influential and highly cited articles in the field that were not listed in the highly cited article [68–79].

## Conclusions

Growth of publications in outdoor air pollution – related respirator health is rapidly increasing. However, limited research output and international collaboration were seen in world regions such as the Middle East, Africa, and South-East Asia. International multidisciplinary research network, involving countries with high levels of air pollution and limited resources, are needed. Research in atmospheric pollution should also be directed toward prevention of air pollution problems by investing more in green technology.

The results presented in this study are indicative of how research activity is interacting with the urgent acceleration of the air pollution crisis at the global level. Furthermore, the research activity is indicative of the response rate adopted by certain countries to face this global problem in a responsible way. Pressure groups can use the research activity to enforce certain environmental and industrial agendas on politicians and political campaigns. Countries with high levels of outdoor air pollution, and therefore, poor air quality, need to get engaged in research pertaining to this field to provide health policymakers with baseline data for future action. Establishing research center for monitoring national air quality and level of air pollution is a step forward that needs to be adopted by all countries. Such centers could include scientists from different disciplines who can collaborate to convert research findings into national agendas and policies. At the national levels, different world countries need to adopt strict guidelines for air quality. Collaboration between industry and health authorities is needed to implement measures that could significantly reduce the levels of particulate matter. The outdoor air pollution is a global public health and therefore research networking between developed countries and developing countries with high levels of air pollution should be prioritized. The Chinese model in controlling air pollution and minimizing its health consequences could be of a global benefit. Finally, since the respiratory effects of air pollution are affecting children, there is a need to educate and increase the awareness of parents regarding this issue.

## Additional file

Additional file 1: Research strategy with keywords. (DOCX 17 kb)

## Abbreviations

IARC: International Agency for Research on Cancer
IRB: Institutional Review Board
WHO: World Health Organization
