# Supplementary material for: Outdoor air pollution and respiratory health: a bibliometric analysis of publications in peer-reviewed journals (1900 – 2017)
Source: Multidiscip Respir Med. 2018 Jun 1;13:15. doi: 10.1186/s40248-018-0128-5 (PMC5984296; doi:10.1186/s40248-018-0128-5)
Supplement: Supplementary file 1 — Research strategy with keywords. (DOCX 17 kb) [file 40248_2018_128_MOESM1_ESM.docx]

**Outdoor air pollution and respiratory health: A bibliometric analysis of publications in peer - reviewed journals**

**Additional file 1**: Research strategy with keywords

| **Step** | **Search query** | **Results** |
| --- | --- | --- |
| **1** | ( ( TITLE-ABS-KEY( "pollut*" AND air ) AND TITLE ("ground level ozone" OR "surface ozone" OR mercury OR "heavy metal*" ) ) OR (TITLE(environment*) AND TITLE-ABS(pollut* or "particul*" or "air quality")) | **6866** |
| **2** | TITLE(air AND pollution) | **21994** |
| **3** | ( ( TITLE ( "air pollution"  OR  "partic* matter"  OR  "fine particulate"  OR  particulate  OR  ozone  OR  "nitrogen *oxide*"  OR  "sulfur *oxide*"  OR  "carbon monoxide"  OR  "air pollutant"  OR  "atmospheric pollut*"  OR  "ultra-fine partic*"  OR  "ultrafine partic*"  OR  "traffic-related pollut*"  OR  "bad air quality"  OR  "atmospheric Lead"  OR  "airborne lead"  OR  "partic* pollut*"  OR  "vehicle emission*"  OR  "road related pllut*"  OR  traffic  OR  "volatile organic gases"  OR  "organic gases"  OR  "outdoor pollut*"  OR  "Ambient air pollution"  OR  "urban air pollut*" )  AND  TITLE-ABS-KEY ( "air pollut*" ) ) ) | **54747** |
| **4** | TITLE-ABS ( "Carbon monoxide " OR pm10 OR pm2.5 OR so2 OR no2 OR o3 ) AND TITLE-ABS ( "air pollut*" ) | **7001** |
| **5** | TITLE ( asthma* OR bronchitis OR respiratory OR "wheez*" OR "pulmonary" OR lung OR emphys* OR copd OR expiratory OR spiromet* OR bronch* ) | **881586** |
| **6** | (1 OR 2 OR 3 OR 4) AND 5  ( TITLE ( asthma*  OR  bronchitis  OR  respiratory  OR  "wheez*"  OR  "pulmonary"  OR  lung  OR  emphys*  OR  copd  OR  expiratory  OR  spiromet*  OR  bronch* ) )  AND  ( ( TITLE-ABS-KEY ( "air pollut*" )  AND  TITLE ( lead  OR  "ground level ozone"  OR  "surface ozone"  OR  mercury  OR  "heavy metal*" ) )  OR  ( TITLE ( air  AND  pollution ) )  OR  ( ( ( TITLE ( "air pollution"  OR  "partic* matter"  OR  "fine particulate"  OR  particulate  OR  ozone  OR  "nitrogen *oxide*"  OR  "sulfur *oxide*"  OR  "carbon monoxide"  OR  "air pollutant"  OR  "atmospheric pollut*"  OR  "ultra-fine partic*"  OR  "ultrafine partic*"  OR  "traffic-related pollut*"  OR  "bad air quality"  OR  "atmospheric Lead"  OR  "airborne lead"  OR  "partic* pollut*"  OR  "vehicle emission*"  OR  "road related pllut*"  OR  traffic  OR  "volatile organic gases"  OR  "organic gases"  OR  "outdoor pollut*"  OR  "Ambient air pollution"  OR  "urban air pollut*" )  AND  TITLE-ABS-KEY ( "air pollut*" ) ) ) )  OR  ( TITLE-ABS ( "Carbon monoxide "  OR  pm10  OR  pm2.5  OR  so2  OR  no2  OR  o3 )  AND  TITLE-ABS ( "air pollut*" ) ) ) | **4361** |
|  |  |  |
| **7** | TITLE ( "ozone depletion" OR "ozone hole" OR "ozone layer" OR "strato* ozone" OR smok* OR in-door OR indoor OR home OR house OR rat OR mice OR mouse OR animal OR experiment* OR pig OR dog* OR horse* OR vetr* OR birds or “cig* smok*”) | **3698** |
| **8** | Exclude 2018 |  |
| **9** | Limit to journal source, exclude errata documents |  |
| **overall** | (( TITLE ( asthma* OR bronchitis OR respirat* OR "wheez*" OR "pulmonary" OR lung OR emphys* OR copd OR expiratory OR spiromet* OR bronch* OR airway*) ) AND ( ( TITLE-ABS-KEY( "pollut*" AND air ) AND TITLE ("ground level ozone" OR "surface ozone" OR mercury OR "heavy metal*" ) ) OR (TITLE(environment*) AND TITLE-ABS(pollut* or "particul*" or "air quality")) OR ( ( ( TITLE ( "air pollution" OR "partic* matter" OR "fine particulate" OR particulate OR ozone OR "nitrogen *oxide*" OR "sulfur *oxide*" OR "carbon monoxide" OR "air pollutant" OR "atmospheric pollut*" OR "ultra-fine partic*" OR "ultrafine partic*" OR "traffic-related pollut*" OR "bad air quality" OR "atmospheric Lead" OR "airborne lead" OR "partic* pollut*" OR "vehicle emission*" OR "road related pllut*" OR "volatile organic gases" OR "organic gases" OR "outdoor pollut*" OR "Ambient air pollution" OR "urban air pollut*" ) AND TITLE-ABS-KEY ( "air pollut*" ) ) ) ) OR (TITLE(traffic OR truck OR road* OR motor OR vehicle) AND TITLE(pollut*)) OR ( TITLE-ABS ( "pm10" OR pm2.5 OR so2 OR no2 OR o3 ) AND TITLE-ABS( "air pollut*" ) ) ) AND NOT TITLE ( "ozone depletion" OR "ozone hole" OR "ozone layer" OR "strato* ozone" OR smok* OR in-door OR indoor OR home OR house* ) AND NOT TITLE-ABS ( "endogenous nitrous" OR nitrosothiol* OR rat OR mice OR mouse OR animal OR "cell culture" OR pig OR "cultured cell" OR dog* OR horse* OR vetr* OR birds) ) AND NOT TITLE-ABS-KEY("cig* smoking") AND ( LIMIT-TO ( SRCTYPE,"j " ) ) AND ( EXCLUDE ( DOCTYPE,"er " ) ) AND ( EXCLUDE ( PUBYEAR,2018 ) ) | **3105** |
